# A Spatial Variation Analysis of In-Hospital Stroke Mortality Based on Integrated Pre-Hospital and Hospital Data in Mashhad, Iran

**DOI:** 10.34172/aim.2023.46

**Published:** 2023-06-01

**Authors:** Eisa Nazar, Habibollah Esmaily, Razieh Yousefi, Jamshid Jamali, Kavian Ghandehari, Soheil Hashtarkhani, Zahra Jafari, Mohammad Taghi Shakeri

**Affiliations:** ^1^Psychiatry and Behavioral Sciences Research Center, Addiction Institute, Mazandaran University of Medical Sciences, Mazandaran, Iran; ^2^Orthopedic Research Center, Mazandaran University of Medical Sciences, Sari, Iran; ^3^Department of Biostatistics, School of Public Health, Social Determinants of Health Research Center, Mashhad University of Medical Sciences, Mashhad, Iran; ^4^Student Research Committee, Mashhad University of Medical Sciences, Mashhad, Iran; ^5^Department of Biostatistics, School of Health, Mashhad University of Medical Sciences, Mashhad, Iran; ^6^Neurocognitive Research Center, Department of Neurology, Mashhad University of Medical Sciences, Mashhad, Iran; ^7^Center for Biomedical Informatics, Department of Pediatrics, University of Tennessee Health Science Center, Memphis, USA; ^8^Clinical Research Development Unit, Ghaem Hospital, Mashhad University of Medical Sciences, Mashhad, Iran

**Keywords:** Bayesian, In-hospital Mortality, Iran, Sequelae, Spatial survival model, Stroke

## Abstract

**Background:**

Despite significant advances in the quality and delivery of specialized stroke care, there still persist remarkable spatial variations in emergency medical services (EMS) transport delays, stroke incidence, and its outcomes. Therefore, it is very important to investigate the possible geographical variations of in-hospital stroke mortality and to identify its associated factors.

**Methods:**

This historical cohort study included suspected stroke cases transferred to Ghaem Hospital of Mashhad by the EMS from March 2018 to March 2019. Using emergency mission IDs, the pre-hospital emergency data were integrated with the patient medical records in the hospital. We used the Bayesian approach for estimating the model parameters.

**Results:**

Out of 301 patients (142 (47.2%) females vs. 159 (52.8%) males) with a final diagnosis of stroke, 61 (20.3%) cases had in-hospital mortality.Results from Bayesian spatial log-logistic proportional odds (PO) model showed that age (PO=1.07), access rate to EMS (PO=0.78), arrival time (evening shift vs. day shift, PO=0.09), and sequelae variables (PO=9.20) had a significant association with the odds of in-hospital stroke mortality (*P*<0.05). Furthermore, the odds of in-hospital stroke mortality were higher in central urban areas compared to suburban areas.

**Conclusion:**

Marked regional variations were found in the odds of in-hospital stroke mortality in Mashhad. There was a direct association between age and odds of in-hospital stroke mortality. Hence, the prognosis of in-hospital stroke mortality could be improved by better control of hypertension, prevention of the occurrence of sequelae, increasing the access rate to EMS, and optimizing shift work schedule.

## Introduction

 Most studies regarding in-hospital mortality are on different illnesses and find that mortality is a common potential critical condition. Stroke is one of the common causes of disability and fatality worldwide.^1^ Despite advances in managing stroke and recent declines in its mortality rates, it is still the third cause of death after heart disease and cancer.^2,3^ According to the World Health Organization (WHO), different rates of stroke mortality are represented in different countries.^4^ While in-hospital stroke mortality rate is around 3–11% in industrial countries,^5^ it is around 7–15% in developing countries.^6^ Iran is a middle-income country and recent reports have shown that the prevalence of stroke in Iran, especially in the young population, is significantly higher than Western countries.^6,7^

 Studies have shown that patients treated in a stroke center usually have a higher survival rate and better health outcomes.^3^ But stroke mortality is known to be also associated with other factors like the patients’ individual factors, quality of pre-hospital emergency medical services (EMS), and hospital cares and facilities.^8^ For example, some studies reported that in-hospital mortality increased in higher ages^5^ and it was significantly higher among females compared to males.^9^ The hospital-related factors include the availability of a specialist stroke center or stroke care unit because these centers play an important role in improving stroke outcomes by designing fine-tuned standards for stroke care and treatment.^7,10^ In addition, pre-hospital delay is a factor related to the quality of pre-hospital EMS, since it affects the timing of stroke treatment.^11^

 In acute stroke management, time of stroke treatment is a very important factor, since a patient loses typically about 1.9 million brain cells for each minute delay in treating ischemic stroke.^12^ In this regard, studies have shown that when intravenous thrombolytic treatment is received within three hours of the onset of symptoms, the outcomes of stroke are improved. So, pre-hospital time, in-hospital time, and appropriate health facilities are important factors in reducing the related mortality rate.^13,14^

 Patients with suspected stroke are usually transferred to the hospital by EMS. EMS is one of the most important kinds of health services in countries, and studies have shown that appropriate pre-hospital care and time of transfer to hospitals are considered to be the most important features in mortality management.^15^ The handling of stroke patients’ treatment varies in different countries, and even between different regions of a city. While many models of stroke care have been described in the literature,^5,6,16,17^ survival analysis considering the geographic situation of patients has been almost ignored.

 The analysis of time until an event of interest, called survival data, has received a great deal of attention in recent decades. This event may be for example death, recovery or disease incidence, and survival time is usually defined in days, weeks, or years. In this analysis, the key point is that the event will not be experienced by all individuals within the given period and some cases are censored.^18^ In survival analysis, data are often collected over distinct spatial locations; so, there is the potential for spatial variations of survival time. Hence, it is substantial to add a spatial component in the survival model for comprehending the variation of an event of interest. The spatial survival models that capture correlation structures have become more common due to computational advances in recent years.^19^ Several recent studies have shown that a low socioeconomic status could influence the risk of stroke,^20-22^ but debate continues over the differences of stroke occurrence in spatial distributions.

 It is essential to investigate possible spatial variations in in-hospital stroke mortality after accounting for known subject-specific pre-hospital and hospital prognostic factors such as access rate to EMS, delay time, revealed access, age, sex, etc. Therefore, it seems that some factors should be of major significance for overall improvement of stroke patients’ situation and reducing mortality rate. Thus, the main purpose of this study is to identify the spatial variation of in-hospital mortality and its associated factors based on integrated pre-hospital and hospital data using Bayesian spatial survival models among stroke patients transferred by EMS.

## Materials and Methods

###  Data Collection

 First, the data on admitted suspected stroke cases from March 2018 to March 2019 were obtained from the EMS system database of the Mashhad City Emergency Management Center (MCEMC). Then, using the emergency mission IDs, the data of patients with a suspected stroke transferred to Ghaem Hospital by the EMS were selected and integrated with the patient medical records in the hospital. As a tertiary neurological referral center, all neurology emergency care is supplied at the Neurology Unit of Ghaem Hospital in Mashhad, which is a megacity in northeastern Iran and the second most populated city of Iran with a population of more than three million people (according to the 2016 census). Finally, out of 2538 patients with suspected stroke transferred to Mashhad hospitals by EMS, we selected 1,284 patients transferred to Ghaem Hospital by the EMS. Then, 301 patients with a final diagnosis of stroke (ICD-10 code: I63.0 to I63.9 and I69.4)^23^ were screened and included in the study.

 Survival time was calculated based on the length of stay (LOS) in the hospital for all patients with a final diagnosis of stroke admitted to the emergency department and hospitalized in different wards for at least one day. So, we excluded all outpatients from the study. We also collected other critical information, including the patient’s condition at discharge (recovered/improved, not recovered, death as an event of interest, transferred to another hospital, escape from the hospital), gender, age, triage level, screening time (hour), arrival time (day shift: 8:00 AM to 4:00 PM; evening shift: 4:00 PM to midnight; night shift: midnight to 8:00 AM), hypertension, and sequelae. In addition, we received the baseline EMS (pre-hospital) information, including the time interval between receiving an emergency call and sending an ambulance (delay time), the time interval between receiving an emergency call and the arrival of an ambulance to the scene (response time), transport time, and callers’ locations from the MCEMC. Also, the sum of response time and transport time was defined as revealed access. Using Google maps, the residential addresses at admission (callers’ locations) were geocoded to latitude and longitude format (X, Y coordinate), which indicated that some patients were from outside of the city of Mashhad. Since our spatial analysis was localized on Mashhad, only patients residing inside Mashhad City were included in the study. Furthermore, access rate to EMS (per one million inhabitants) was calculated by the two-step floating catchment area (2SFCA) method.^24-30^ This approach captures the interactions between ambulance availability and the population distribution, generating an access score for each of the census blocks through a two-step process:

 Step 1: For each EMS station *j*, we searched all population locations (*k*) (population of the neighborhoods) within a threshold travel time (*d*_0_ = 10 min) from station *j* (i.e. the catchment of this EMS location), and computed the provider-to-population ratio *R*_j_ within the catchment area using equation 1:


(1) $$R_{j}=\frac{S_{j}}{\sum_{k\in \left\{d_{kj}\leq d_{0}\right\}}P_{K}}$$

where *P*_k_ is the population at location *k* where the centroid falls within catchment *j* (*d*_kj_ ≤ *d*_0_), *S*_j_ the number of ambulances at station *j*, and *d*_kj_ the travel time between *k* and *j*.

 Step 2: For each census block centroid *i*, we searched all station locations (*j*) within the threshold travel time (*d*_0_ = 10 min) from location *i*(i.e. the catchment area), and summed up the provider-to-population ratios (derived in step 1) *R*_j_ at these locations using equation 2:


(2) $$A_{i}^{F}=\sum_{j\in \left\{d_{ij}\leq d_{0}\right\}}R_{j}$$

where $A_{i}^{F}$ represents the accessibility of the population at block *i* to EMS ambulances based on the 2SFCA method, *R*_j_ the provider-to-population ratio at station location *j* where the centroid falls within the catchment area centered at population location *i* (i.e. *d*_ij_ ≤ *d*_0_), and *d*_ij_ is the travel time between *i* and *j*.

 The averages of access scores of census blocks in each neighborhood were calculated and visualized so as to yield a better interpretation and comparison of the results. We used the Jenks natural break method to classify the index into five classes. This method seeks to reduce the variance within classes and maximize the variance between classes.^31^

###  Statistical Analysis

 To describe the continuous and qualitative variables mean ± standard deviation (SD) and frequency (%) were reported, respectively. To compare the baseline characteristics of patients and pre-hospital information by mortality, a univariate Cox regression model was applied. Then, variables with *P* < 0.30 were entered into the final multiple models.

 For the survival-time analysis, a log-logistic proportional odds (PO) model was used to assess the associated factors with in-hospital mortality of stroke patients. This model was chosen because the proportional hazard assumption in the Cox PH model is violated based on the Schoenfeld residuals. The probability density function of log-logistic distribution with two parameters (b is a shape parameter, and λ is a positive scale parameter) is given by equation 3:^32^


(3) $$f\left(t\left|b,\lambda \right.\right)=\frac{\lambda bt^{b-1}}{{\left(1+\lambda t^{b}\right)}^{2}}\;,\;t>0,\;b>0,\;\lambda >0$$

In addition, patient’s residence can also impact survival as it is often associated with common risk factors of disease (such as air pollution, access to healthcare, and socio-economic factors), which may affect stroke outcomes such as in-hospital mortality. Hence, despite increasing advances in the quality and delivery of specialized stroke care, spatial variations in stroke outcomes continue to persist. Accordingly, geographical referencing is substantial in the comprehension of variations in in-hospital mortality of stroke patients. Also, we considered a Bayesian spatial log-logistic PO model with Gaussian random field (GRF) priors for analyzing pre-hospital and hospital stroke data. It is noteworthy that the survival and density functions of PO model are according to equation 4:


(4) $$S_{x_{ij}}\left(t\right)=\frac{e^{-x_{ij}^{T}\beta -\upsilon_{i}}S_{0}\left(t\right)}{1+\left(e^{-x_{ij}^{T}\beta -\upsilon_{i}}-1\right)S_{0}\left(t\right)}\;,\;f_{x_{ij}}\left(t\right)=\frac{e^{-x_{ij}^{T}\beta -\upsilon_{i}}f_{0}\left(t\right)}{{\left[1+\left(e^{-x_{ij}^{T}\beta -\upsilon_{i}}-1\right)S_{0}\left(t\right)\right]}^{2}}$$

where $\beta ={\left(\beta_{1},\beta_{2},...,\beta_{p}\right)}^{T}$ is a vector of regression coeﬃcients, $\upsilon_{i}$ is an spatial frailty associated with location exactly observed *s*_i_, *s*_i_ is a location on the surface area under study defined by latitude and longitude format (X, Y coordinate), and *S*_0_(*t*) is the baseline survival function with density *f*_0_(*t*) corresponding to *x*_ij _= 0 and $\upsilon_{i}=0$ .^33^

 In Bayesian framework, we considered the following prior distributions for each parameter in the model:


$$\begin{array}{l} \beta ∼N_{p}\left(\beta_{0},S_{0}\right)\\ S_{0}\left(.\right)\left|\alpha ,\theta ∼TBP_{L}\left(\alpha ,S_{\theta }\left(.\right)\right),\alpha ∼\Gamma \left(a_{0},b_{0}\right),\theta ∼N_{2}\left(\theta_{0},V_{0}\right)\right.\\ {\left(\upsilon_{1},\upsilon_{2},...,\upsilon_{m}\right)}^{T}\left|\tau ,\phi ∼GRF\left(\tau^{2},\phi \right),\tau^{-2}∼\Gamma \left(a_{\tau },b_{\tau }\right),\phi ∼\Gamma \left(a_{\phi },b_{\phi }\right)\right. \end{array}$$

Where *TBP*_L_ and GRF are transformed Bernstein polynomial (TBP) prior^34,35^ and GRF prior, respectively.

###  Gaussian Random Field Priors

 In georeferenced data (location exactly observed), it is generally supposed that $\upsilon_{i}=\upsilon \left(S_{i}\right)$ arises from a GRF { $\upsilon \left(S\right)$ , s ∈ S} such that $v=\left(\upsilon_{1},\upsilon_{2},...,\upsilon_{m}\right)$ follows a multivariate Gaussian distribution as $v∼N_{m}\left(0,\tau^{2}R\right)$ , where $\tau^{2}$ measures the amount of spatial variations across locations and the (i, j) element of R is modeled as $R\left[i.j\right]=\rho \left(s_{i},s_{j}\right)$ . Here, $\rho \left(.,.\right)$ is a correlation function controlling the spatial dependence of $\upsilon \left(s\right)$ and the powered exponential correlation function $\rho \left(s,s^{\prime }\right)=\rho \left(s,s^{\prime };\phi \right)=\exp\left\{-{\left(\phi \left\|s-s^{\prime }\right\|\right)}^{\upsilon }\right\}$ is used, where ϕ>0 is a range parameter controlling the spatial decay over distance, υ∈(0,2] is a shape parameter, and $\left\|s-s^{\prime }\right\|$ is a Euclidean distance between location *s* and $s^{\prime }$ .^33^

 Cox-Snell plots were used to assess the fit of a model, and the model choice was made via deviance information criterion (DIC) and Watanabe–Akaike information criterion (WAIC). Both criteria are readily calculated from the MCMC output with 15 000 burn-in and 15 000 iterations. Lower values of DIC and WAIC indicate an appropriate fit of the model. Furthermore, trace plots were used for assessing the convergence. All analyses were performed using SPSS version 20 and package spBayesSurv^33^ in R statistical software version 3.6.2 at the significance level of 0.05.

## Results

**Figure 1:**
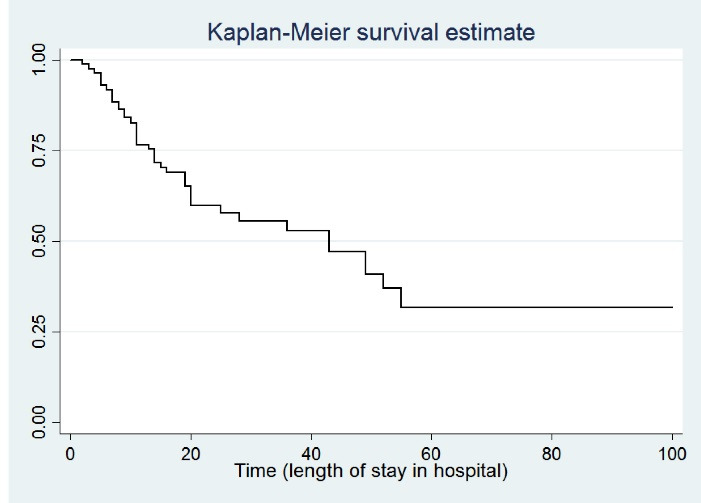
Plots of Kaplan-Meier Product Limit Estimates of Survival of Stroke Patients

 Out of 301 patients with a final diagnosis of stroke, 61 (20.3%) cases had in-hospital mortality, and 240 (79.7%) patients were censored and experienced other discharge statuses such as recovered, personal satisfaction, transferred to another hospital, escape from the hospital, and follow-up treatment. Table 1 demonstrates the demographic and EMS call characteristics of stroke patients from March 2018 to March 2019 in Mashhad. Also, 142 (47.2%) of patients were female with a mean age of 69.9 ± 14.2, while 159 (52.8%) were male with a mean age of 71.4 ± 13.2. Figure 1 shows the estimated survival probabilities of stroke patients using the Kaplan-Meier method, and the median survival time was 43.0 ± 9.4 days.

**Table 1:** Baseline and EMS Calls Characteristics of Stroke Patients (n = 301)

**Characteristics**	**Overall**	**In-hospital mortality**	
		**No **	**Yes **
**Hospital Information**			
Age (y)			
Mean ± SD	70.7 ± 13.7	69.6 ± 13.3	75.0 ± 14.3
< 63	85 (100)	74 (87.1)	11 (12.9)
≥ 63	216 (100)	166 (76.9)	50 (23.1)
Gender			
Female	142 (100)	118 (83.1)	24 (16.9)
Male	159 (100)	122 (76.7)	37 (23.3)
Triage level			
Level 1	2 (100)	0 (0.0)	2 (10.0)
Level 2	218 (100)	167 (76.6)	51 (23.4)
Level 3	79 (100)	71 (89.9)	8 (10.1)
Level 4	2 (100)	2 (100.0)	0 (0.0)
Screening time (h)	0.16 (0.13-0.24)	0.16 (0.13-0.23)	0.16 (0.13-0.25)
≤ 0.20	198 (100)	159 (80.3)	39 (19.7)
> 0.20	103 (100)	81 (78.6)	22 (21.4)
Arrival time			
Day shift	140 (100)	108 (77.1)	32 (22.9)
Evening shift	108 (100)	89 (82.4)	19 (17.6)
Night shift	53 (100)	43 (81.1)	10 (18.9)
HTN			
No	95 (100)	79 (83.2)	16 (16.8)
Yes	206 (100)	161 (78.2)	45 (21.8)
Sequelae			
No	289 (100)	231 (79.9)	58 (20.1)
Yes	12 (100)	9 (75.0)	3 (25.0)
**Pre-hospital Information**			
Delay time (second)	29.0 (21.0-47.0)	29.0 (21.2-47.0)	31.0 (20.0-45.5)
≤ 30	160 (100)	130 (81.3)	30 (18.8)
> 30	141 (100)	110 (78.0)	31 (22.0)
Reveal access (min)	31.6 ± 13.9	31.6 ± 13.7	31.6 ± 14.6
Response time (min)	9.0 ± 3.8	9.1 ± 3.8	8.8 ± 3.9
Transport time (min)	20.0 (14.5-27.2)	20.0 (14.7-27.4)	20.4 (13.4-25.8)
Access rate to EMS services (per 1 million)	27.0 ± 7.2	27.3 ± 6.8	26.0 ± 8.7

HTN: Hypertension. Values are reported as frequency (percent), mean ± SD or median (Q1–Q3).

**Figure 2:**
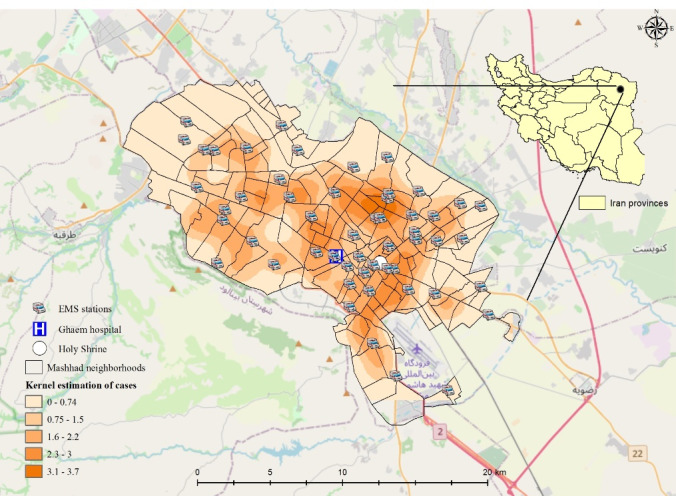
Geographical Distribution of Stroke Patients, EMS Stations, Ghaem Hospital, and Point Density Map of Estimated Frailty Values of Each Patient According to the Spatial Model in Mashhad, Iran

**Figure 3:**
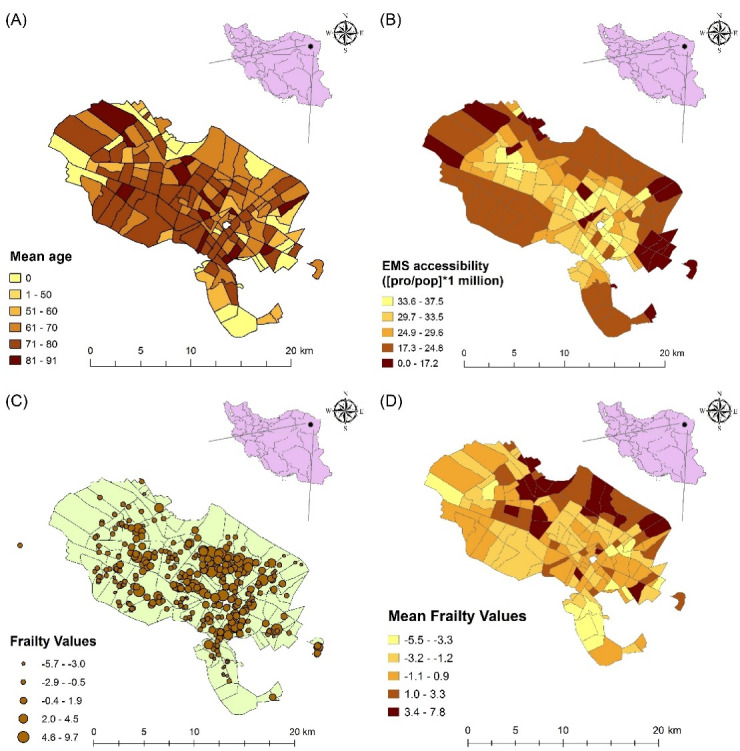
(A): Mean age of patients with final diagnosis of stroke in the Mashhad districts; (B): Access rate to EMS services (per 1 million inhabitants) in the Mashhad districts; (C): Estimated frailty values from the Bayesian spatial log-logistic PO model with the GRF prior for each patient; (D): Estimated frailty values from the Bayesian spatial log-logistic PO model with the GRF prior in each district

Figure 2 shows the location of Ghaem Hospital and EMS stations in the study area, Mashhad, Iran. According to Figure 3A, the mean age of patients in the southwestern districts of Mashhad was higher than in other districts. Figure 3B shows the access rate to EMS services (per 1 million inhabitants) in Mashhad districts. As the figure suggests, due to the huge aggregation of EMS stations in the city center zones, the access rate to EMS services in the suburban areas is lower than central urban areas.

 Results from the univariate cox regression model revealed that the arrival time (evening shift vs. day shift with HR (95% CI) = 0.56 (0.31, 1.008), *P* = 0.04) and sequelae (HR (95% CI) = 4.39 (1.35, 14.30), *P* = 0.01) variables had a significant association with the hazard of in-hospital mortality in stroke patients (Table 2). Then, variables with *P* < 0.30, including age (*P* = 0.06), revealed access (*P* = 0.29), access rate to EMS service (*P* = 0.23), delay time (*P* = 0.15), triage level (*P* = 0.24), arrival time (evening shift and night shift vs. day shift with *P* = 0.04 and *P* = 0.21, respectively), HTN (*P* = 0.24), and sequelae (*P* = 0.01) entered into the multiple log-logistic PO models. The goodness of fit criteria presented in Table 3 suggested that the Bayesian spatial log-logistic PO model had better performance compared to the Bayesian non-spatial log-logistic PO model (DIC = 430.1 and WAIC = 438.4 vs. DIC = 441.9 and WAIC = 452.9). The results from our selected model showed that such variables as age (PO (95% CI) = 1.07 (1.01, 1.17)), access rate to EMS services (PO (95% CI) = 0.78 (0.63, 0.94)), arrival time (evening shift vs. day shift with PO (95% CI) = 0.09 (0.002, 0.86)), and sequelae (PO (95% CI) = 9.20 (1.08, 172.43)) had a significant association with the odds of in-hospital stroke mortality. Furthermore, by adjusting the effect of other variables, the odds of in-hospital mortality in patients with sequelae was 9.20 times higher than those patients without sequelae. Also, by adjusting the effect of other variables, the odds of in-hospital mortality in patients admitted during evening shifts compared to day shifts was 0.09 times, while the odds of in-hospital mortality in patients admitted during the night shift was 0.14 times compared to day shifts. Furthermore, for each unit increase in the age, after adjusting for the effects of other variables, the odds of in-hospital mortality of stroke patients increased by 7%. Also, for each unit increase in the access rate to EMS services (per 1 million inhabitants), after adjusting for the effect of other variables, the odds of in-hospital mortality of stroke patients decreased by 22%. In addition, by controlling the effect of other variables, the odds of in-hospital mortality with abnormal hypertension was 4.8 times higher than those with normal hypertension, which was not statistically significant. Other variables had no significant association with the odds of in-hospital mortality in stroke patients (Table 3).

**Table 2:** The Results from of the Univariate Cox Regression Model

**Characteristics**	**HR (95% CI)**	***P *****value**
Age	1.01 (0.99, 1.03)	0.06
Reveal access	0.99 (0.98, 1.01)	0.29
Access rate to EMS services	0.98 (0.95, 1.02)	0.23
Delay time	1.003 (0.99 1.007)	0.15
Gender		
Female	Ref	
Male	1.24 (0.74, 2.09)	0.41
Triage level		
Levels 3 & 4	Ref	
Levels 1 & 2	1.42 (0.66, 3.01)	0.24
Arrival time		
Day shift	Ref	
Evening shift	0.56 (0.31, 1.008)	0.04^*^
Night shift	0.74 (0.36, 1.51)	0.21
Screening time (hour)		
≤ 0.20	Ref	
> 0.20	0.94 (0.56, 1.60)	0.84
HTN		
No	Ref	
Yes	1.41 (0.79, 2.50)	0.24
Sequelae		
No	Ref	
Yes	4.39 (1.35, 14.30)	0.01^*^

HTN, Hypertension; CI, confidence intervals; HR, hazard ratio; *significant at the level of 0.05.

**Table 3:** Results of the Spatial and Non-spatial (Frailty) Log-logistic PO Model

**Characteristics**	**Non-spatial Log-logistic PO model** **PO (95% CI)**	**Spatial Log-logistic PO model** **PO (95% CI)**
Age	1.04 (0.99, 1.09)	1.07 (1.01, 1.17)^a^
Reveal access	0.96 (0.87, 1.05)	0.96 (0.87, 1.02)
Access rate to EMS services	0.88 (0.78, 0.99)^a^	0.78 (0.63, 0.94)^a^
Delay time	1.007 (0.99 1.02)	1.002 (0.99, 1.02)
Triage level		
Levels 3 & 4	Ref	Ref
Levels 1 & 2	1.95 (0.12, 21.32)	1.50 (0.16, 19.10)
Arrival time		
Day shift	Ref	Ref
Evening shift	0.07 (0.006, 0.73)^a^	0.09 (0.002, 0.86)^a^
Night shift	0.14 (0.006, 4.66)	0.14 (0.002, 2.55)
HTN		
No	Ref	Ref
Yes	5.41 (0.55, 84.77)	4.80 (0.53, 75.18)
Sequelae		
No	Ref	Ref
Yes	6.68 (0.99, 51.93)	9.20 (1.08, 172.43)^a^
DIC	441.9	430.1
WAIC	452.9	438.4

HTN, Hypertension; CI, confidence intervals; HR, hazard ratio; PO, Proportional Odds. ^a^ The credible interval does not include one and is significant at the level of 0.05.

**Figure 4:**
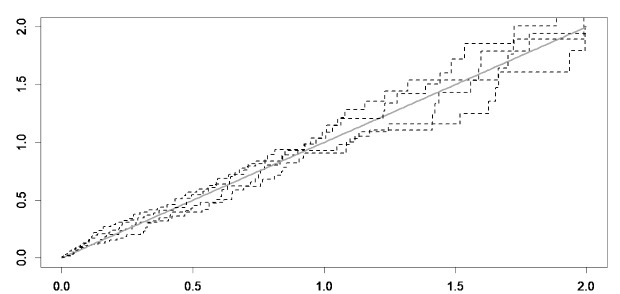
Cox-Snell Plot of Spatial Log-logistic PO Model

**Figure 5:**
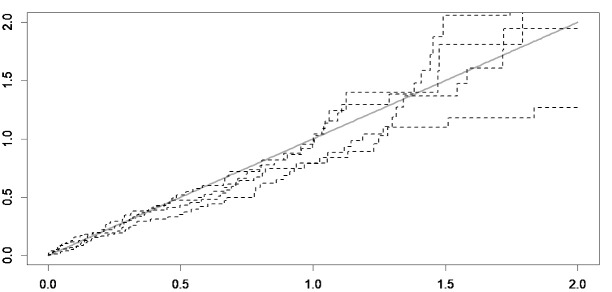
Cox-Snell Plot of Non-spatial (frailty) Log-logistic PO Model

Figure 3C shows the map of frailty values of each patient estimated by the Bayesian spatial survival model with GRF priors, where the higher estimated frailty values indicate higher odds of in-hospital mortality. In addition, the point density analysis^36^ of these estimated frailty values is depicted in Figure 2. As can be seen, the odds of in-hospital mortality in the central urban areas was higher than suburban areas, since higher values indicate higher odds of in-hospital stroke mortality. Also, the average estimated frailty values of each district were calculated and visualized in Figure 3D. As shown, there is higher odds of in-hospital stroke mortality in central urban and north-eastern areas compared to other regions. Also, through considering the Cox-Snell plot and comparing the jagged line with the reference line (45◦ lines), it can be observed that the Bayesian spatial log-logistic PO model had a better fit for these data compared to the Bayesian non-spatial log-logistic PO model (Figures 4 and 5).

## Discussion

 The main objective of this research was to evaluate the performance of EMS and people’s access rate to EMS (per 1 million inhabitants) in different regions of Mashhad, as well as its relationship to the spatial variations of in-hospital stroke mortality. Our results revealed that the access rate to EMS (per 1 million inhabitants) in the suburban areas was lower compared to central urban areas due to the huge aggregation of EMS stations in the central area. In this regard, our results were compatible with the findings of some other studies^30,37,38^; this implies that there is a remarkable spatial pattern in scene-to-hospital arrival time, call-to-hospital arrival time, and access rate to EMS in Mashhad. Therefore, health policymakers should pay attention to the balanced distribution of EMS stations, especially in areas with high gaps and low access rates to EMS. Meanwhile, since central areas of the cities have heavier traffic compared to the suburbs, it seems that constructing special EMS roads is necessary on high-traffic city center routes. The results of the present study showed that 20.3% of patients had in-hospital stroke mortality, which is in line with the findings of the study by Matuja et al,^39^ and higher than deaths reported by Alhazzani et al.^8^

 Our results from the selected model (suggested by the goodness of fit criteria), as the Bayesian spatial log-logistic PO model, revealed that growing age significantly increased the odds of in-hospital mortality (PO = 1.07). This finding is in agreement with the results of a recent systematic review which found a significant positive correlation between age and the mortality rate.^8,40^ The present study revealed that hypertension increases the odds of in-hospital mortality in stroke patients (PO = 4.80). This result was supported by findings from these studies.^8,41-43^ Also, our study confirmed the positive association between sequelae and odds of in-hospital stroke mortality (PO = 9.20). Moreover, our study showed a positive association between delay time and odds of in-hospital stroke mortality, so that for each minute increase in delay time, the odd of in-hospital stroke mortality increased by PO = exp(60*0.002) = 1.12. However, there was no statistically significant difference, which can be related to the small sample size. Hence, it is recommended to conduct studies with larger sample sizes. Due to the important role of EMS in health systems of all communities, the first-line measure in fatality management is to provide appropriate pre-hospital care and timely transfer of patients to hospitals.^15,44-46^ In our study, the EMS response time and revealed access was 9.0 ± 3.8 and 31.6 ± 13.9, respectively. However, in a different study conducted in Rasht in northern Iran, the EMS response time and revealed access was reported to be 6.5 and 26.5 minutes. In another research carried out in Yazd in central Iran, the EMS response time was within eight minutes in approximately 80% of cases. Nevertheless, it is notable that both Rasht and Yazd have significant differences in terms of the road traffic condition and total population compared to Mashhad.^47,48^ Also, increasing the access rate to EMS significantly decreased the odds of in-hospital stroke mortality (PO = 0.78).

 Our findings showed that the odds of in-hospital stroke mortality (spatial frailty values estimated by Bayesian spatial log-logistic PO model) differed across various locations, such that the odds of death in central parts of urban areas, despite the higher level of access to EMS, was higher than in the suburbs; this is in line with the results of these studies.^8,49,50^ These variations may be related to differences in different regions in terms of incidence, mortality, or both.^51^ Additionally, the reported differences might be attributed to such factors as high-density population, heavy traffic, and high rate of air pollution despite the fact that the access rate to EMS was greater in central urban areas. In addition, the number of older individuals in central areas of cities is usually higher than in the suburbs. Therefore, it is recommended that more research should be conducted on this area by considering the limitations of the present study, including the effect of traffic, population density, percentage of elderly people in each district based on census information, and air pollution. Another limitation of our study is the existence of sparse-data bias in some variables such as sequelae that may be due to the relatively small sample size in our study.

 This main strength of this study is its internal validity based on the statistical analysis conducted and the quality of gathered data. Using emergency mission IDs, we integrated data obtained from the EMS system database of City Emergency Management Center with patient medical records in the Ghaem Hospital. Also, the presence of the exact residential addresses of patients helped us to estimate the odds of in-hospital stroke mortality in any observed location. This data provides a comprehensive account of the stroke in Mashhad districts, which may show the same in any given area over any given period. The spatial survival model can well capture the local residual spatial dependence, and it can be the representative of the studied territory for effective management of stroke in Mashhad.

 In conclusion, in Mashhad, the odds of in-hospital stroke mortality were higher in central districts of the city compared to the suburbs. Our results also showed that there were disparities in access rate to EMS in Mashhad, and the odds of in-hospital stroke mortality increased with age. It is assumed that the odds of in-hospital stroke mortality could be significantly reduced by controlling hypertension in a better way, prevention of the occurrence of sequelae, increasing the access rate to EMS, and optimizing shift work scheduling. Indeed, to improve the management of outpatients and also reduce their referral to hospitals, expert EMS team members could be trained. These findings might help researchers and local health policy-makers that seek to reduce disparities in health services and in-hospital stroke mortality.
